# Key challenges in simulated patient programs: An international comparative case study

**DOI:** 10.1186/1472-6920-11-69

**Published:** 2011-09-25

**Authors:** Debra Nestel, Diana Tabak, Tanya Tierney, Carine Layat-Burn, Anja Robb, Susan Clark, Tracy Morrison, Norma Jones, Rachel Ellis, Cathy Smith, Nancy McNaughton, Kerry Knickle, Jenny Higham, Roger Kneebone

**Affiliations:** 1 Gippsland Medical School, School of Rural Health, Faculty of Medicine, Nursing and Health Sciences, Monash University, Northways Road, Churchill, Victoria, 3842, Australia; 2 Standardized Patient Program, University of Toronto, 88 College Street, Toronto, ON M5G 1L4, Canada; 3 Department of Surgery & Cancer, Imperial College London, Praed Street, London, W2 1PD, UK; 4 University of Applied Sciences Health Sciences, Lausanne (HECVSanté), Av. de Beaumont 211011 Lausanne, Switzerland; 5 The Harry Partnership, 159 Green Lanes, London, N16 9DB, UK; 6 Department of Family and Community Medicine, University of Toronto, 500 University Avenue, 5th Floor, Toronto, M5G 1VF, Canada; 7 Faculty of Medicine, Imperial College London, Exhibition Road, London, UK, SW7 2AZ

## Abstract

**Background:**

The literature on simulated or standardized patient (SP) methodology is expanding. However, at the level of the program, there are several gaps in the literature. We seek to fill this gap through documenting experiences from four programs in Australia, Canada, Switzerland and the United Kingdom. We focused on challenges in SP methodology, faculty, organisational structure and quality assurance.

**Methods:**

We used a multiple case study method with cross-case synthesis. Over eighteen months during a series of informal and formal interactions (focused meetings and conference presentations) we documented key characteristics of programs and drew on secondary document sources.

**Results:**

Although programs shared challenges in SP methodology they also experienced differences. Key challenges common to programs included systematic quality assurance and the opportunity for research. There were differences in the terminology used to describe SPs, in their recruitment and training. Other differences reflected local conditions and demands in organisational structure, funding relationships with the host institution and national trends, especially in assessments.

**Conclusion:**

This international case study reveals similarities and differences in SP methodology. Programs were highly contextualised and have emerged in response to local, institutional, profession/discipline and national conditions. Broader trends in healthcare education have also influenced development. Each of the programs experienced challenges in the same themes but the nature of the challenges often varied widely.

## Background

Simulation-based education is expanding rapidly in medical and other health professional curricula. In the United Kingdom (UK), simulation was recently identified by the Chief Medical Officer as one of five significant challenges for health services in the next decade [1]. The drivers for the expansion of simulation-based education are well documented including ethical imperatives, working time directives, patient empowerment, patient safety movement and the need for learner-centred education [2-5]. The latter is compromised in real clinical settings where there is inherent tension between the primary need for clinicians to be patient-centred yet also respond to trainees in a learner-centred way [6]. It is not possible to be both patient- and learner-centred in the same space. Simulation permits learner-centredness while remaining patient-focused [7].

In this paper, we examine SP programs drawing examples from Australia, Canada, Switzerland and the United Kingdom (UK). We use the terms 'simulated' and 'standardised' patient (SP) to describe a person trained to portray a patient in a clinical scenario for educational purposes. The SP may also provide feedback to students, trainees and clinicians on their performance. In Australia and the UK, 'simulated patient' is usually a generic term where 'standardised patient' refers to actors trained to reproduce an equivalent performance for students in high stakes assessments [8]. In Canada and Switzerland, the term 'standardised patient' refers to all SP-based activities. In this paper we use the terms interchangeably. We use the term 'trainee' when referring to any learner interacting with an SP unless describing specific cohorts.

SPs have potential to be the highest fidelity 'simulator' and are well established in most undergraduate medical programs [9,10]. Their contribution is growing in pharmacy, nursing and other health professions [11-13]. There is also a heightened interest in patient perspectives being offered at all stages of medical and health professional curriculum design. That is, from curriculum planning through development, implementation and evaluation. There is also acknowledgement that patients often experience health care differently to those who deliver it [14-16]. Although real patients have much to contribute to clinical education [17-19], there are also challenges associated with their direct involvement in teaching. Real patients, may be unwilling or unable to contribute, cannot be 'standardised' for a sustained period and may feel compromised in their relationship with the clinicians caring for them. SPs, as a proxy for real patients offer significant advantages [9,10,20,21]. SPs can allow systematic delivery of curriculum instead of more opportunistic learning in clinical settings. The provision of detailed constructive feedback to students from SPs is a feature of SP contribution to student learning. Reasons for the uptake of SP-based education are listed in Table 1.

**Table 1:** Drivers for uptake of simulated patient-based education

• Raised profile of patient perspectives and patient empowerment
• Ethical imperative of causing no harm to patients
• Implementation of working time directives
• Prominence of the patient safety movement
• Increased numbers of medical and health professional students
• Reduced hospital stays for patients
• Growing evidence of simulation as an effective educational method
• Growing evidence that effective health professional/patient communication is key to patient and clinician (learner) satisfaction and reduces litigation
• Development of national assessments
• Facilitates a systematic approach to curriculum activities
• Development of 'professional' competencies
• Carefully constructed simulations
○ Assure students have direct/indirect exposure
○ Allow for adjustment in the level of challenge
○ Identify boundaries of competence
○ Provide access to technical, communication and other professional skills essential for safe clinical practice
○ Enable rehearsal of infrequently occurring events
○ Assure the development of reflective practice (video, debriefing)

There are several reviews on different facets of SP methodology [10,22,23], valuable descriptive papers and other resources [24,20]. However, the literature reveals gaps in several aspects of SP methodology, especially at the level of the program [9,25-29]. That is, the organisational unit of SP programs. This case study attempts to bridge these gaps by addressing the following questions:

1. What are key challenges associated with SPs in SP programs?

2. What are key challenges for faculty in SP programs?

3. What are key challenges in organisational structure of SP programs?

4. What are key challenges of quality assurance in SP programs?

## Methods

We adopted a multiple case study approach [30-32] suited to uncovering contextual conditions relevant to the research questions. Over eighteen months in formal and informal rounds of discussion the authors met intermittently to report and compare experiences in SP methodology across four programs in universities in Australia, Canada, Switzerland and the UK. We used secondary sources of data for characterising the SP programs and documented discussions in textual notes (e.g. summary statistics from databases, policy and curriculum documents). After establishing program characteristics, we address each of the research questions adopting the analytic method of cross-case synthesis. In this process, individual cases are first documented and then compared across cases. We sought to identify similarities and differences between programs in relation to specific challenges.

## Results

Our experiences with SP programs are summarised according to number of SPs, recruitment strategies, training programs, principal foci, funding models, pay, research output, future plans and challenges (Table 2).

**Table 2:** Summary of simulated patient programs

	Gippsland Medical School	University of Toronto	University of Applied Sciences, Section Health Lausanne	Imperial College London
Program established	2008	1984	2009	1998

Numbers of SPs on register	45	592 > 100 core teaching > 500 for examinations	38	260

Recruitment strategy	Personal referral Amateur dramatic group Information on website Registration online	Personal referral/word of mouth Complete application Personal interview by two program members or small group interview Application file No advertising	Personal referral/word of mouth Articles in specialised newsletters Performing art school First selection (phone call) Personal interview (1.5 hour) by one/two program member(s) including a role-play	Register online Web-based information 'Screening' through half day program once a year Referral through SPs, educators, specialised actor agencies (e.g. The Harry Partnership) and other London medical schools

Training program	Minimum 2-hour generic introductory SP training Before teaching and assessment sessions Focused SP training (including rating and feedback)	Minimum 2-hour training, brush-up or dry-run for teaching and assessment assignments Feedback workshops	Minimum 5-hour training for teaching and assessment activities Focused SP training (including rating, oral feedback and working with tutors) Feedback workshop once a year	New SPs may undergo training - minimum 2-hour training Usually before teaching sessions Before assessment sessions Focused SP training (e.g. working with tutors, working with student tutors, feedback)

Principal focus	Teaching and assessment of medical students, international medical graduates and other health professionals in the region Participation in research projects Quality assurance	Teaching and assessment of medical students, pharmacy students, international medical graduates Continuing education Faculty development Research projects National licensing examinations Quality assurance	Teaching and assessment of health professional students (radiologic technology, nursing, physiotherapy and midwifery) Participation in research projects Continuing education Quality assurance	Teaching and assessment of medical students Participation in research projects (especially in piloting innovative approaches to simulation based procedural and operative skills training) Quality assurance

Funding model	University funded for teaching and assessment undergraduate students Specific projects funded by government, research bodies or commissioning institutions	Fee for service Hourly rate charged 25% access fee & mark up to cover benefits paid to SPs and to cover all administrative salaries & operating expenses	University funded for teaching and assessment undergraduate students Specific projects funded by government, research bodies or commissioning institutions	University funded for teaching and assessment of medical students Specific projects funded by government, research bodies or commissioning institutions

Hourly pay	~AUD23 per hour 3 hour minimum	CdnD15-25 per hour depending on assignment CdnD12 per hour to train 2 hour minimum	CHF 30 per hour for teaching without feedback and assessment CHF 40 per hour for teaching with an oral/written structured feedback	GBP30 per hour for teaching GBP25 per hour for exams Non-role players for physical examination GBP75 examination session ~GBP150 per day 3 hour minimum

Research	Roles and responsibilities of SPs; Theoretical underpinning from performing arts and theatre studies; SP-based education for international medical graduates	Patient focused simulation for procedural skills; Collaborative learning; Sensitive communications (e.g. error disclosure, bioethics); Changing scope of practice in pharmacy and family medicine; Emotional impact on simulation participants; Role of SPs in assessment; Unannounced SPs in clinical practice; Roles and responsibilities of SPs	Effects of SP feedback; Roles and responsibilities of SPs in health professional training	Patient-focused simulation for procedural and operative skills; Training methods for patient-focused simulation; Expanding the function of SPs to role-play health professionals

Future plans	Expand to other health care professions Develop a model for SP programs in regional and rural locations Expand the university wide database (MonSim) Develop a paediatric SP program Develop and extend resources that reflect local health service needs (e.g. indigenous SPs, mental health, disaster recovery, international medical graduates) Develop flexible training resources for online learning Promote quality assurance of program	Increase work with international medical graduate specialties Expand to business and law Develop teaching and learning resources (e.g. instructive/trigger videos) Develop SP database for on-line communication, bookings, payroll, etc.	Extend the SP pool Promote quality assurance of program Develop patient focused simulation Develop research projects Develop teaching and learning resources (e.g. demonstration, instructive/trigger videos) Share resources with medical institutions in Lausanne	Improve co-ordination across College Share resources with central London medical schools Continue research program

### Gippsland Medical School, Monash University, Churchill, Australia

The SP program at Gippsland Medical School was developed in 2008 to support graduate entry medical students in a new medical school at Monash University. There are sixty to ninety students in each of the four years of the medical curriculum. Gippsland is situated in a rural environment and is characterised by high levels of community engagement. There are 45 SPs on the database. The program has academic leadership, is closely associated with a clinical skills curriculum theme and has technical and administrative support. SPs support the development of clinical skills in medical students with an emphasis on communication. They work almost exclusively with first-year students. SPs make a substantial contribution to implementation of skills assessments in the Objective Structured Clinical Examination (OSCE). The School has facilities designed to support teaching and learning in simulated clinical settings with opportunities for audiovisual review.

### University of Toronto, Toronto, Canada

As the largest (SPs = 592) and oldest, the SP program at the University of Toronto was started in 1984 in the Department of Family and Community Medicine (DFCM). As its activities expanded beyond family medicine, the program moved to the Wilson Centre for Research in Education. The program director reports to the Education Dean responsible for Continuing Education and Professional Development. SP teaching and assessment is thoroughly embedded in medical and health professional curricula. The cost-recovery program has eighteen full-time staff who manage academic and business activities. There have been enormous changes in the program - from the initial typed SP roles kept in a single filing cabinet and hand-entered accounts in a small black ledger the program has evolved to web-based training materials and an electronic database to manage over 500 registrants. SPs are employees of the University and classified as casual members of the United Steelworkers of America (USWA). Exponential growth during the 1990s was largely in response to the performance-based component of Canadian national licensing examinations in medicine, pharmacy and physiotherapy. The focus of the program has been teaching and learning clinical skills with an emphasis on communication behaviours. SP-based activities have expanded to include standardised caregivers/relatives, standardised students for faculty development, standardised parties in dispute for conflict resolution and standardised health professionals for inter-professional and team training.

### University of Applied Sciences, HECVSanté, Lausanne, Switzerland

The SP program of HECVSanté was developed in 2009 and is located in the Unit of Educational Innovation. The SP program supports activities in four faculties - nursing, midwifery, physiotherapy and radiology technology. The program has an academic lead, an SP trainer (50%) and an administrator (20%) who work closely with the clinical skills centre. The database has 38 SPs. Each course has 25 to 38 students per year. SPs support the acquisition of clinical skills with an emphasis on communication. SP feedback to students plays a critical role in the educative process. The SP program has a strong interprofessional orientation.

### Imperial College, London, United Kingdom

At Imperial College, the SP program evolved in 1998 with the creation of a Faculty of Medicine, an amalgamation of three established undergraduate medical schools together with research and other institutes in central London. The College graduates approximately 350 students each year. There are 260 SPs on the Imperial database. SPs work throughout the medical curriculum for teaching and assessment in a range of sessions on communication, general practice, patient safety and ethics. Although there is one SP program (recruitment, training, teaching, assessment, and research) located in the Department of Surgery and Cancer, there are several databases across the College. Similarly, there are several administrators supporting local needs with one overall academic lead.

### Summary of SP databases

Table 3 summarises the characteristics of the SPs on the program databases. For each program there are more females than males. Although the mean age at Toronto is lower than the other programs, this is because five per cent of the SPs are under sixteen years of age. Between seventeen and twenty per cent of SPs at Imperial and Toronto speak languages other than English reflecting the communities in which the programs are located. The first language of all SPs in Lausanne is French.

**Table 3:** Summary data of simulated patients on databases

	Gippsland Medical School	University of Toronto	Lausanne	Imperial College
Male	22	257	9	120

Female	36	332	29	140

Mean age	50	38	45	50

Age range	10 - 84	12 -89	20-75	19 - 65

Under 16 years	1	30	None	None

First language	100% (English)	97% (English)	99% (French)	98% (English)

Fluent in other languages	3%	17% (French, Spanish, Italian, German, Greek, Cantonese, Portugese, Tamil)	2%	20% (French, German, Spanish, Italian, Dutch, Maltese, Russian, Kutchi, Cantonese, Teochow, Japanese, Mandarin, Punjabi, Hungarian, Arabic and others)

British Sign Language/Makaton/Other	0	0	0	3%

### 1. What are key challenges associated with simulated patients in SP programs?

Many challenges emanated from SPs and the nature of their work. We discuss these according to recruitment, categories of SPs and performance and training.

#### Recruitment

Challenges vary between programs with under and oversubscription influencing recruitment strategies. At Toronto there are 153 applicants awaiting interviews. At Imperial there is a waiting list to join the SP program while Gippsland and Lausanne are undersubscribed. Criteria for selection to SP programs are intentionally broad at Gippsland and Imperial. That is, SPs need to be interested, reliable and have an ability to retain information. After expressing interest, SPs at Gippsland are invited to a training session in which the methodology is outlined and demonstrated. While some participants withdraw at this point, the remainder stay on the database and are contacted when a session is scheduled. Personal and program referral (other medical schools) are the most common methods for recruitment at Imperial. In collaboration with other medical schools, Imperial offer recruitment and training sessions enabling SPs to demonstrate their role-play and feedback skills before joining the program. At Toronto, applicants are put through a rigorous screening and selection process that involves a large group orientation, followed by interview with at least two SP representatives. At Lausanne, SPs are also screened. After an initial phone interview, the SP attends the centre where they are interviewed, complete a questionnaire addressing different facets of SP work including their reasons for participating and have an opportunity to demonstrate their role-playing skills. All SPs have a 'conditional period' before being admitted to the program.

Substantial gaps exist in all four programs with respect to special populations (e.g. ethnicity, age groups, sex) requiring targeted advertising or recruitment by referral.

#### Categories of simulated patients

The programs have several categories of SPs summarised in Table 4. The different categories create challenges in management such as the different contractual relationship associated with volunteers and paid SPs. Effective SP program management will ensure that the most appropriate category of SP is resourced to meet the needs of the trainees in the planned session. At Gippsland and Imperial volunteer SPs write roles based on their own experiences and in their own words. This process generates a large number of authentic roles in a short period [33]. However, they may not always address trainees' needs. Volunteer SPs support novice students with self-reported benefits for students and SPs. Professional SPs participate in teaching, assessment and research. Unlike volunteers, professional SPs are able to take on any number of roles. At Imperial and Toronto, most SPs have had formal actor training since London and Toronto have relatively large performing arts communities. Gippsland and Lausanne rely almost exclusively on SPs who have no performance studies or actor training.

**Table 4:** Categories of simulated (standardised) patients

1. Volunteers (Gippsland, Imperial):
a. Play a role that is based on their own experience [36]
b. Are drawn from local community
c. Are unpaid
d. Have different levels of training in case portrayal and feedback skills
2. Physical examination role players (Imperial, Lausanne):
a. Play a given role with minimal speaking since the focus is on assessment of students in physical examination
b. Are drawn from local community/drawn from clinical cases which showed learning obstacles for students
c. Are paid
d. Have different levels of training
3. SPs (Gippsland, Imperial, Toronto, Lausanne):
a. Play roles in teaching, assessments and research
b. May or may not have formal acting or performance training
c. Undergo training as SPs in case portrayal and feedback skills
d. Are paid an established hourly rate

#### Performance and training

The sessional nature of SP work means that under-performing SPs (e.g. late, inconsistent, forgetful or unprofessional) may not be invited for further work. Although training is always considered as an option to improve performance, the nature of the performance deficit will determine the course of action. At Toronto and Lausanne, teachers working with SPs are asked to complete brief written evaluations on scenario realism, SP performance and quality of feedback. Further, processes have been developed to note areas of concern with reference to SP performance. If an extreme breach of professional behaviour occurs, the SP is released (e.g. taking a mobile phone call during a high stakes assessment).

All four programs provide training that outlines learning objectives for trainees, the schedule, expectations of the SP and an opportunity for reviewing and rehearsing roles. Sessions on feedback provide guidance to SPs on the preferred format and process. Feedback varies according to the session goals and may include the use of structured protocols and rating forms. Feedback may be in-person and immediately after the role-play or videotaped for later review by the trainee. Training sometimes includes tutors with whom the SPs will be working but this is often difficult to schedule. Training for assessments includes examination protocols (e.g. staying 'in role', confidentiality, negotiating judgements etc), expected trainee standards, rehearsal and calibration of performance.

At Gippsland, SP training consists of generic programs that introduce the concept of SP-based education. These are offered up to twice annually to people who have expressed interest in being an SP. At Toronto, with its established cohort of SPs, the program has developed an annual professional development day which offers a menu of workshops on a variety of topics from which attendees (~200) can choose.

### 2. What are key challenges for faculty in SP programs?

We define faculty as all staff associated with SP programs - administrators, educators, clinicians and academics. A key challenge for all four programs is faculty development. That is, supporting faculty in further developing expertise in their roles.

At Gippsland, Imperial and Lausanne, the SP program leads have academic appointments. They also have responsibilities outside the SP program as communication, clinical and/or education specialists. The leads provide training to SPs and tutors and share responsibility for SP role development and curriculum design. Research activity is expected and is perceived to be fundamental to success. Faculty development for academic staff is supported through attendance at conferences and other professional meetings. Tutors are offered focused training on working with SPs.

At Imperial and Toronto, as part of quality assurance, a set of responsibilities for all those involved in SP work was developed through stakeholder consultation [34]. The document sets out expectations of the program director/lead, tutors, SPs, students and administrators. The guidelines have been adopted at Gippsland and provide clarity for all those involved in SP-based work. Previously there has been no clear career trajectory for SPs. Entry points to such careers have been opportunistic. Further, little attention has been paid to succession planning.

Historically, the Toronto program has been considered a 'service' entity although clearly it has become an integral part of the academic enterprise at the University. A project manager/SP trainer takes charge of a given course or assignment. Although the SP program is working toward full faculty appointments, this process is complicated by the union presence. Research activities and attendance at international conferences are strongly encouraged.

### 3. What are key challenges in organisational structure of SP programs?

The challenges associated with organisational structure varied according to scale of the program. The relationship of the program to the host institution is critical together with management and leadership.

#### Relationship with host institution

At Gippsland and Lausanne, the SP program's relationship with the host institution is very clear. The program exists to support trainees in the school/faculty. At Imperial, the SP program resides in one department although it works across the Faculty of Medicine. Again, it exists largely to support the delivery of the medical curriculum. At Toronto, the program operates as a business with a cost-recovery funding model based on fee for service. Although the program started in medical education, it now works across several faculties and outside healthcare. The program charges higher rates for private (commercial) enterprises, which partially underwrites university projects.

#### Management and leadership

At Gippsland, the clinical skills team provide leadership. The medical school offers administrative support. Academic activities include curriculum development, SP training program development, delivery and research. Administration support activities include maintenance of a database, recruitment and booking of SPs, preparing rooms and clinical skills equipment, audiovisual management, arranging payment, ensuring human resource compliance and copying program materials. As a relatively new program there is an opportunity to implement robust structures and processes for SP program management (e.g. writing, storing and retrieving roles; templates for role development etc).

At Toronto, the director, who is also the professional manager, oversees the program. There are three associate directors with portfolios of research, academic and business, a manager of SP relations and program operations, a systems manager, two business administrative officers, a digital media specialist, and nine faculty who function as project managers, SP educators, session leaders and SP trainers. All employees with the exception of the director are classified as Union members (USWA). This is an important point of difference with the other programs where union membership is not compulsory.

At Lausanne, the SP program has a manager and academic leader both of whom work closely with discipline-specific teachers to develop relevant SP-based activities. The academic lead has responsibility for the expansion of the program and research. Administration activities include development and maintenance of a database, copying materials, establishing contracts, arranging payment, booking rooms, and organising evaluations.

At Imperial, there is an SP academic lead who has responsibility for the program across the medical curriculum. This allows close ties with academics in several departments enabling a multi-disciplinary approach to writing scenarios. Administrators manage the SP database and all human resource support. The recent formation of the Imperial Academic Health Science Centre through the merging of two West London Hospital Trusts and Imperial College has added an extra level of complexity to the organisation, but has encouraged collaboration between academic and clinical teams.

#### Funding models

Funding models play a significant role in SP programs. The global financial crisis has had significant impact. The teaching and assessment activities at Gippsland, Imperial and Lausanne are funded through university budgets allocated to the curriculum. SP payments fall within this budget as do staff who support the program. Research projects and educational activities outside the curriculum are paid independently by the funding source. At Toronto, the cost recovery model has been in place since the outset of the program. There is a fee schedule that includes a mark up to cover Toronto benefits paid to SPs and an "access fee" of 25% covering the salaries of full-time staff and all operating expenses. SPs are provided to any programs within and outside of the University for which fees are paid.

#### Contractual arrangements

SPs at Gippsland sign one-year contracts prior to undertaking work. There is one rate of pay for all SP activities (i.e. training, teaching, assessment, research, resource development etc). Pay forms are completed at the time of the session with a minimum number of three hours for each session.

At Imperial and Lausanne, SPs are employed on a sessional basis for specific teaching, assessment and research. Fees vary depending on the purpose of the session. At Imperial, SPs do not sign a contract and are paid according to an hourly rate, with a minimum payment of three hours for a session. Most SPs are registered as self-employed, and are required to provide proof of self-employment. Because London has several medical schools geographically close together, many of the SPs also work regularly for other medical schools.

At Toronto, SPs do not sign contracts. However, they are required to read and acknowledge a letter setting out responsibilities and expectations of the SP and the program. SPs are classified as casual employees and are therefore members of the USWA. Fees vary depending on the complexity of the session with a minimum payment of two hours.

#### Databases

SP databases contain information about SPs that enable appropriate casting. Examples of content contained in the databases are included in Table 5. Gippsland have developed a Faculty-wide web-based database enabling access for schools of nursing, and allied health professionals. Administrators can search the database by the variables. After selection, the database generates session specific information to the designated SP in an email.

**Table 5:** Content of simulated (standardized) patient databases

Name Title Date of birth Current age Email Telephone Address Physical description Body shape Height Weight Scars - face, abdomen, chest, back, limbs Facial features - beard, moustache Hair - bald, length, colour Body hair - chest Special features - glasses, tattoos, piercings, hearing aid, pace maker	Level of education Languages spoken Sign language Willing to participate in: Teaching Research Exams/Assessments Physical examination - minimal exposure, partial undress Intimate examination - e.g. breast Roles suited to play Roles trained for Roles played Confidentiality agreement and disclaimer

At Imperial and Lausanne, the database is currently in Microsoft Excel, with plans to move to a web-based system to increase flexibility. At Imperial, SPs are sourced and booked electronically. The large scale of examinations (with some exams occurring simultaneously over multiple sites) has facilitated change with more electronic systems used for confirmation of bookings. However, this program requires further development. Currently only the program manager and administrators have access to the database, acting as gatekeepers to other interested parties.

Toronto faces several challenges in data management and has survived several phases of re-design. Financial records and payroll must be compatible with the larger university system. The main database and some supplementary databases unique to outside clients are contained in Microsoft Access providing complete information on every SP. Although progress has been made towards booking and communicating with SPs electronically, not all SPs are computer literate. The program website will become the forum for all SP communication.

### 4. What are key challenges of quality assurance for SP programs?

All SP programs recognised the need for quality assurance at the level of the program. This includes setting standards for recruitment, training and feedback processes. Gippsland and Lausanne implemented processes to capture session evaluations. This was considered especially important as they are relatively new programs with evolving resources. Their scale also permitted collection and collation of data. Toronto and Imperial are more established and have targeted session evaluations. Each program had informal measures in place to identify under-performing SPs. Faculty are encouraged to report any concerns to program leads who will decide how best to deal with the issue. Programs acknowledged the need to improve feedback to SPs on all aspects of their work. Although valued by program directors and SPs, feedback to SPs is often omitted because of time pressures and hourly rates of pay.

## Discussion

Local (e.g. access to professional actors), institutional (e.g. amalgamation of schools, funding models, commitment to simulation, innovation in education), discipline/profession (e.g. uni-, multi- or inter-professional) and national (e.g. assessments, scale) contexts have profoundly influenced the development of the SP programs. The four programs have emerged in different educational 'eras' and to meet different needs. Although highly contextualised, there are similarities between programs. These include their shared goal to provide high quality educational opportunities for trainees. Recent sharing of resources between programs has facilitated standard setting and perceived enrichment of programs. However, funding models constrain the extent to which resources can be freely shared.

While each program faced similar challenges the nature and direction of challenges differed. For example, in synthesizing key challenges for SPs, recruitment proved an issue. Two programs had difficulty recruiting SPs (Gippsland and Lausanne) while two programs were oversubscribed (Imperial and Toronto). This led to different recruitment strategies and selection processes. However, all programs were challenged by recruitment of special populations (e.g. ethnic groups, different age groups, males). Similarly, with performance and training, there were differences in the nature of challenges. The amount and type of training was strongly influenced by program resources and the volume of SP work.

In the synthesis of key challenges for faculty in SP programs, faculty development was highly valued and well supported for academic leads but there was less evidence of development opportunities for other faculty. For key challenges in the organisational structure of SP programs, clarity in the relationship with the host institution was critical and influenced several facets of the programs. The programs differed in relation to the type of leadership. That is, academic or professional management. The nature of the leadership in part reflected the funding models for each program. In this case study, only Toronto had a cost-recovery model. That is, they are self-funded charging for all services. In contrast, the three other programs were funded through university curriculum budgets. Additional differences between programs related to the role of union membership. Again, only Toronto had a union presence.

We hypothesise that the program 'culture' is influenced by their core business and scale. For example, the 'program' at Gippsland does not have an identity independent of its host institution while the program at Toronto has an international profile. The culture of the SP program at Toronto is highly 'professional'. The program sustains many SPs in work on a weekly basis. In contrast, at Gippsland and Lausanne no individual SP works on a weekly basis. This reduces opportunities for development and advancement of the methodology because there are fewer opportunities to practice, Further, undersubscribed programs are more likely to be challenged by quality assurance issues associated with the limited pool of SPs. It is also apparent that SP programs take time to 'mature' as SPs develop expertise through practice, feedback and mentoring [35] and are able to take on more sophisticated tasks.

In this analysis, national assessments and scale are interrelated. In our case study, only Canada has national health professional exams at the undergraduate level. This strongly influenced the size and focus of SP programs. Identity was also influenced by the location of the program in the University - in a small medical school, as an independent SP program, in specialist education unit and in a surgical department.

Terminology between SP programs varied and this created confusion and misunderstanding in cross-institutional comparisons. Notable differences in programs include their longevity, the consequent level of experience of SPs, numbers of registrants, funding models, pay rates, research profile and challenges. Research activity was partially influenced by funding models. However, local champions and expertise in educational research was also important.

The SP programs at Gippsland, Imperial and Toronto support the development of patient-centred communication and other professional skills in medical students and doctors. Lausanne differs in its target group working almost exclusively across professional disciplines. Toronto also works extensively across health professional groups. Imperial and Toronto cater to large numbers of trainees. In each program, strong academic and administrative leadership was considered critical for embedding SP-based education in curricula and for seeking new opportunities.

The literature offers little guidance on recruitment although qualities of SPs are regularly reported [11,24]. Quality assurance was highly valued but an area for significant development in each program.

The Gippsland and Imperial programs are co-located with clinical skills simulation centres. Broader trends in the 'accreditation of simulation centres' are likely to impact SP programs. SPs in these programs were perceived as core to such centres rather than separate.

SP programs need to be responsive to local needs. Although larger and more established programs can offer a suite of educational activities, they were also constrained by instructional and regulatory issues. They have the ability to offer large-scale examinations while smaller programs focused on meeting specific local needs. The scale of the program seems to create different types of challenges.

### Limitations of the approach

The authors were all involved in the research process and so it is possible that responses were biased. However, the extended period over which the study occurred and the multiple authors provided repeated opportunities for validation. Further, it is possible that as a self-selected group of academics sharing a similar philosophy to SP methodology there is bias in our reporting. The cases do not represent all types of SP programs. We acknowledge the highly contextual nature of each SP program and like much qualitative research we do not make claims for generalisability.

## Conclusions

Using a case study approach we have offered insight to four SP programs in different parts of the world. We specifically examined key challenges in SP methodology, of faculty in SP programs, of organisational structure and quality assurance. While each program faced similar challenges the nature and direction of challenges differed. For example, programs were challenged by SP recruitment with over and under subscription, which in turn led to different recruitment strategies and selection processes.

As simulation-based education expands we hope our experiences may be valuable for faculty entering this field and for those already established, to promote reflection on their programs and to plan future activities. We do not make recommendations about 'best practice' because our experiences demonstrated that each SP program was highly contextualised. We hope others will take from each program what they think is appropriate for their own. We believe our own practices have improved as a consequence of sharing experiences in the process of preparing this paper. A concluding message is the need for continued efforts in establishing an evidence-base of SP methodology.

## Competing interests

The authors declare that they have no competing interests.

## Authors' contributions

DN and DT developed the concept. All authors contributed to the provision of data for the case study. DN drafted the first manuscript. All authors contributed to the writing of the manuscript. All authors have read and approved the final manuscript.

## Pre-publication history

The pre-publication history for this paper can be accessed here:

http://www.biomedcentral.com/1472-6920/11/69/prepub
